# Co-evolution of *Mycobacterium tuberculosis* and *Homo sapiens*

**DOI:** 10.1111/imr.12264

**Published:** 2015-02-20

**Authors:** Daniela Brites, Sebastien Gagneux

**Affiliations:** 1 Department of Medical Parasitology and Infection Biology, Swiss Tropical and Public Health Institute and University of BaselBasel, Switzerland

**Keywords:** host, pathogen, adaptation, virulence, selection

## Abstract

The causative agent of human tuberculosis (TB), *Mycobacterium tuberculosis*, is an obligate pathogen that evolved to exclusively persist in human populations. For *M. tuberculosis* to transmit from person to person, it has to cause pulmonary disease. Therefore, *M. tuberculosis* virulence has likely been a significant determinant of the association between *M. tuberculosis* and humans. Indeed, the evolutionary success of some *M. tuberculosis* genotypes seems at least partially attributable to their increased virulence. The latter possibly evolved as a consequence of human demographic expansions. If co-evolution occurred, humans would have counteracted to minimize the deleterious effects of *M. tuberculosis* virulence. The fact that human resistance to infection has a strong genetic basis is a likely consequence of such a counter-response. The genetic architecture underlying human resistance to *M. tuberculosis* remains largely elusive. However, interactions between human genetic polymorphisms and *M. tuberculosis* genotypes have been reported. Such interactions are consistent with local adaptation and allow for a better understanding of protective immunity in TB. Future ‘genome-to-genome’ studies, in which locally associated human and *M. tuberculosis* genotypes are interrogated in conjunction, will help identify new protective antigens for the development of better TB vaccines.

## Introduction

Co-evolution between a host and a pathogen occurs when evolutionary changes in the pathogen that increase infectivity are counteracted by evolutionary changes in the host that increase resistance to infection. Such reciprocal changes have been demonstrated in experimental systems such as bacteria and their bacteriophages (1,2). However, in host–pathogen associations with higher levels of biological complexity, a rigorous demonstration of these phenomena is not straightforward (3), particularly when considering infectious diseases of humans (4). Yet, in many instances, despite the lack of a formal proof of host–parasite-induced reciprocal changes, co-evolution still remains a likely explanation for many of the patterns observed in host–pathogen interactions.

For co-evolution to occur between a host and a pathogen: (i) pathogen infectivity and host resistance have to have reciprocal antagonistic effects on the fitness of each; (ii) the variation in pathogen infectivity and in host resistance have to be at least partially genetically determined; and (iii) the outcome of the interactions between the partners has to be dependent on the respective genotypes (5–8). Here, we review the association of *Mycobacterium tuberculosis* complex (MTBC) and *Homo sapiens* in light of these aspects.

Tuberculosis (TB) remains a leading cause of morbidity and mortality in the world (9). Despite years of research, no vaccine currently exists that protects reliably against pulmonary TB in adults, which is the most transmissible form of the disease (10). Indeed, although many components of the host immune response against MTBC are known, the specific molecules and mechanisms underlying protective immunity remain elusive (11,12). Remarkably, some hallmarks of TB infection and disease, such as latency, remain poorly understood (13,14). Evidence is emerging that in addition to host and environmental factors, the genetic variation in MTBC also plays a role in the clinical phenotypes of TB (15,16). However, little is known about the interaction between human and MTBC genetic diversity, and it has been argued that new paradigms and new conceptual frameworks are required to better understand and ultimately better control TB globally (12,14,17,18).

The purpose of this review was to discuss new ideas and concepts that together could guide future research. We focus on the bacterial side of the association, but always in the context of its relation to the host. We start by introducing MTBC, the different phylogenetic lineages that make up this group of organisms, as well as their biogeographical distribution and primary host range. Next, we discuss how MTBC virulence might have evolved in response to different evolutionary forces, in particular human demography. Local adaptation is a phenomenon expected from a co-evolutionary association, and the evidence for it in human TB is presented. Co-evolution is expected to lead to interactions between host and pathogen loci; hence, we summarize the existing evidence with respect to such interactions in TB. Recent research on the age of MTBC is summarized in light of both genetic and archeological data. In the final section of this review, we discuss how genetic drift and natural selection might influence the ability of MTBC to adapt to its host and end by highlighting how understanding more about the co-evolutionary history of MTBC and humans can guide us in our quest for a better TB vaccine.

## The *Mycobacterium tuberculosis* complex

MTBC comprises various bacterial species and sub-species sharing 99.9% DNA sequence identity but differing in their primary host range. We describe below the different members of MTBC, dividing them according to their primary host: human-adapted MTBC lineages, the lineages adapted to wild and domestic mammalian hosts, and *Mycobacterium canettii*. For simplicity, throughout this review, we use the term MTBC to refer to all MTBC lineages except *M. canettii*.

### The human-adapted MTBC lineages

**Figure 1:**
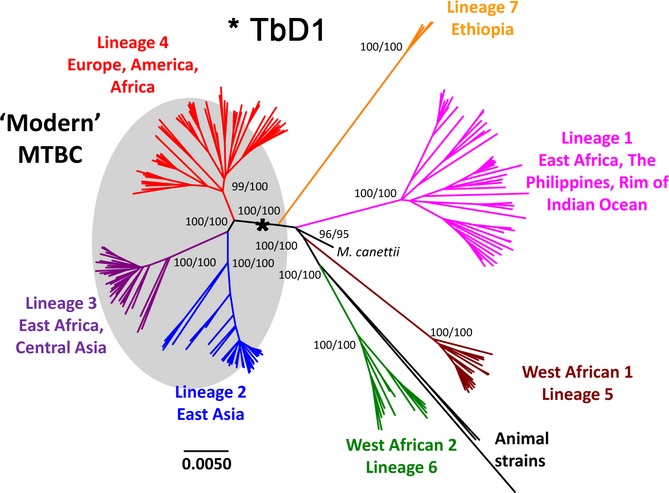
Whole-genome phylogeny of 220 strains of MTBC. Bootstrap support for the main branches after inference with Neighbor-joining (left) and Maximum-likelihood (right) analyses are shown. The grey circle indicates the ‘modern’ strains (see main text). Scale bars indicate substitutions per site. The occurrence of the TbD1 deletion is indicated with an asterisk. Adapted from Comas *et al*. (29).

The human-adapted forms of MTBC include *Mycobacterium tuberculosis sensu stricto* and *M. africanum*. Humans are the only known host where infection and transmission by *M. tuberculosis* and *M. africanum* occur efficiently and where infection cycles are known to be sustainably maintained (19). Unlike postulated until a decade ago, it is now clear that humans did most likely not acquire the tubercle bacilli from cattle during animal domestication, as certain human MTBC lineages are phylogenetically more basal (i.e. ‘ancestral’) than *M. bovis* (20,21). Furthermore, *M. bovis* has lost several genes that are still present in all other *M. tuberculosis* lineages and does not contain any gene that does not occur in at least some strains of *M. tuberculosis sensu stricto*. For a better understanding of the terminology used to classify the main lineages of MTBC, we briefly summarize the discoveries underlying that nomenclature. A main step into elucidating the genetic structure of MTBC was the recognition that a group of strains associated with major epidemics like the ‘Beijing’ and ‘Haarlem’ strains all shared a deletion (TbD1) that is not present in *M. africanum* strains (20). As large-scale ongoing horizontal gene transfer has not been described in MTBC, it was assumed that the TbD1-deleted strains were younger, in evolutionary terms, than strains without this deletion. Accordingly, TbD1-deleted strains have been referred to as evolutionary modern, whereas *M. africanum* and several other MTBC lineages were considered ancient (20,22). It was also recognized that TbD1 was not deleted in *M. bovis* and that *M. africanum*,*M. bovis*, and other animal strains shared another deletion (RD9) not present in the modern strains or any other strains belonging to *M. tuberculosis sensu stricto* (20). During the subsequent decade, the phylogenetic relationships among the different MTBC groups were further deciphered using different genetic markers, culminating in large-scale comparative whole-genome sequencing (23–29). The most recent phylogenetic inferences reveal that the human-adapted lineages of MTBC have a strong phylogeographical structure comprising seven major phylogenetic lineages that diverged from a common ancestor and diversified in different regions of the world (23–28). The TbD1-deleted strains known as modern are now recognized as three separate, albeit related lineages: Lineage 4 (also known as Euro-American) that has a broad distribution in Europe and America but also in Africa and the Middle-East, Lineage 2 (East-Asian lineage, includes the Beijing family of strains) that is widely distributed in East Asian countries, and Lineage 3 that has a relative narrow distribution occurring in East Africa and in Central and South Asia (24,26) (*Fig. *1). In addition to the animal-adapted lineages, the ancient lineages without the deletion in TbD1 comprise Lineage 1 (also known as Indo-Oceanic), which occurs around the Indian Ocean and the Philippines, Lineage 5 (also known as *M. africanum* West African 1), and Lineage 6 (*M. africanum* West African 2), which are geographically restricted to Western African countries (30), and the recently described Lineage 7 (Ethiopian lineage), which is restricted to Ethiopia (24,26,28). Rooting (*Table *1) the phylogenetic relationships between the different MTBC lineages with *M. canettii* reveals that the common ancestor of *M. africanum* Lineages 5 and 6, and the animal-adapted lineages split early from the common ancestor of MTBC. Another early split led to the ancestor from which all lineages belonging to *M. tuberculosis sensu stricto* derived, with Lineage 1 being the most basal within this group of strains, followed by Lineage 7. A later divergence event originated the ancestor of the modern Lineages 2, 3, and 4 (*Fig. *1). Note that the modern Lineages 2, 3, and 4 are monophyletic (*Table *1) and therefore comprise a more closely related group of organisms, compared to the various ancient lineages, which are paraphyletic (*Table *1). The genetic properties of these lineages and the phenotypical and epidemiological consequences of that variation have been reviewed recently elsewhere (15,16). Here, we focus on the aspects we consider most relevant for understanding the evolution of MTBC and its association with different human population. Members of the modern Lineages 2 and 4 are responsible for most TB cases in the world, and certain strains belonging to these lineages have been implicated in major outbreaks of both drug-sensitive and drug-resistant TB (15,16,31). Furthermore, these lineages have been shown to cause faster progression to active disease and to be more virulent in animal models than ancient lineages (reviewed in (15,16)). Their monophyletic origin and their expansion through different parts of the world suggest that they could have accompanied the first migrations of modern humans ‘Out-of-Africa’ and diversified together with different human populations (26,32). Following this scenario, the prediction would be that particular MTBC lineages would be better adapted to their local host population; this seems to be at least partially the case, as further discussed below. It is notorious that certain lineages, but also sub-groups within the main MTBC lineages (we might call those ‘sub-lineages’) are geographically more widespread than others. Whether this geographic population structure reflects local adaptation, human demography, founder effects, or a combination of these remains unclear. Comparing the geographically restricted Lineages 5, 6, and 7 with the geographically much more widespread Lineages 2 and 4 provides good examples. Moreover, some sub-lineages within Lineage 4 also differ in their geographic distribution; for example, the Haarlem and the LAM families occur worldwide, while other sub-lineages are geographically restricted. Examples for the latter include the Cameroon and Uganda families of strains, which are the most prevalent strains those countries, respectively (33,34). Human migrations might account for some of these patterns. For instance, Haarlem and LAM strains were likely brought to the Americas by Europeans during colonization and the massive European migrations of the 18th and 19th centuries to the New World (35). By contrast, even though large numbers of Africans were brought to the Americas during the slave trade, African MTBC variants like *M. africanum*, or the Cameroon and Uganda sub-lineages of Lineage 4 are almost completely absent from the Americas (30). Hence, the differential distribution of MTBC lineages and sub-lineages could also reflect intrinsic characteristics of the strains. For example, some lineages might be thought of ‘generalists’, i.e. able to persist in different human populations, and other more ‘specialists’, i.e. able to persist only in one or a few particular host populations (36).

**Table 1:** Definition of terms used from phylogenetics and population genetics

Term	Definition
Rooting	Providing the evolutionary order of phylogenetic branching events, by defining the position of the common ancestor to all samples on a phylogenetic tree
Monophyletic group	A group that contains all the descendants of a common ancestor
Paraphyletic group	A group that has a common ancestor but that does not included all descendants of that common ancestor
Molecular clock	Amount of differences between the DNA molecules of two species is a function of the time since their evolutionary separation
Calibration point	Attributing one or more dates to tips (e.g. taxa) or nodes of the phylogenetic tree. Dates are usually based on fossils, but they can also be based on known historical events, or in the case of fast evolving microorganism, on isolation times
Coalescent time of a set of strains	The time that has passed since the most recent common ancestor of all those strains existed
Polymorphism	A genetic variant that segregates within a population
Substitution	A genetic variant fixed between two species
Population bottleneck	Reduction on the size of a population, at least for one generation, due to external effects
Founder effect	Establishing a new population by a small number of individuals not genetically representative of the original source population. Leads to loss of genetic diversity by genetic drift
Genetic drift	Random fluctuation of alleles in populations, e.g. after a population bottleneck, randomly, certain alleles are removed, whereas others are kept
Background selection	The effect that selection on deleterious mutations has on other linked non-deleterious mutations, leading to loss of genetic diversity
Positive selection	Selection favoring an advantageous mutation
Negative selection	Selection against a deleterious mutation

In apparent contradiction with the idea of host-specific pathogen adaptation in MTBC is the fact that transmission of *M. tuberculosis* to animals and *M. bovis* to humans does occur occasionally (37,38). This also has occurred in our evolutionary past, as revealed by the detection of ancient *M. bovis* DNA in 2000 years old human bones in Siberia (39) and of DNA from strains related to *M. pinnipedii*, an MTBC member usually found in seals and sea lions, in 1000-year-old human remains from Peru (40). The latter study provided the first whole-genome sequence from ancient MTBC DNA and has been stimulating the discussion on the origin of TB in the Americas (41). There is evidence for Pre-Columbian TB in coastal regions of South America; extant South American MTBC strains, however, were most likely introduced by Europeans, as most of autochthonous TB in that part of the world today is caused by Lineage 4 (24). However, whether the *M. pinnipedii*-related strains could have been a general source of infection in the Americas 1000 years ago and whether these strains were human-adapted remains unknown. Only sequencing more pre- and post-Columbian strains from different geographical regions across the Americas will allow a more definite conclusion.

Although there is some level of cross-species infection and transmission, sustainable human-to-human transmission of animal-adapted MTBC strains has not been demonstrated (42). As we discuss below, the animal strains all derived from one common ancestor that is shared with *M. africanum* Lineage 6, which is currently considered a human-adapted lineage, and with no other MTBC lineage. Given the proximity of domestic animals and humans throughout human evolutionary history, there were certainly opportunities for jumps of other human MTBC lineages to animals. However, this does not seem to have occurred throughout the evolution of MTBC, supporting the notion that the human-adapted MTBC lineages became specialized to infect and persist in human populations.

### The animal-adapted MTBC lineages

In contrast to the human-adapted MTBC lineages, some animal-adapted strains of MTBC are able to establish infections and transmit within other animal species aside from their primary hosts. We briefly discuss the animal-adapted lineages, as their geographic distribution, host range, and virulence characteristics might help us to better understand the association of *M. tuberculosis sensu stricto* and *M. africanum* with humans. The animal-adapted MTBC comprise the following: *M. microti*, found in voles, wood mice, and shrews but recently suggested to have spilt-over to domestic cats in the United Kingdom (43); the so-called dassie bacillus, which is a pathogen of hyraxes (44,45); *M. pinnipedii* found in seals and sea lions; *M. caprae* found in goats but also described as causing sustainable disease in red deer populations (46,47); and *M. bovis* found in cattle but also able to infect other domestic and wild animals, in particular badgers (19,48). Other members of the complex have been described more recently. *M. orygis* has been found in oryxes, gazelles, deer, antelopes, waterbucks, and buffalos, but also in humans from East and Southeast Asia (49,50); indeed, at least half of the *M. orygis* isolates reported to date have come from human TB patients (49). More recently, *M. mungi* was described from mongoose populations in Botswana (51) and *M. suricattae* in meerkats from South Africa (52). Finally, a novel MTBC strain was isolated from a wild chimpanzee in Cote d'Ivoire (31). All these wild and domestic animal lineages of MTBC and the human-adapted *M. africanum* Lineage 6 share the deletions RD7, RD8, RD9, and RD10, supporting the notion that *M. tuberculosis* did not derive from a bovine form during the Neolithic Revolution, as traditionally postulated. These findings also suggest that the MTBC diversity in wild animals may be much greater than previously appreciated. The hypothesis that all these animal-adapted lineages descended from a singly human-adapted ancestor might also be too simplistic (31). Perhaps, the common ancestor of Lineage 6 and all the animal-adapted strains was not primarily human-adapted but rather a promiscuous bacillus with a broad host spectrum. Host specialization might then have occurred at a later stage during the evolution of these lineages. Interestingly, just like in the human-adapted MTBC lineages, the African continent also harbors most of the diversity in animal-adapted lineages, further supporting the African origin of MTBC (24,26,29,53). Another interesting observation is that most of the animal-adapted lineages affecting primarily wild animals remained geographically restricted to Africa, whereas the lineages adapted to domestic animals spread to different parts of the world, possibly as a consequence of human migration and trade.

**Figure 2:**
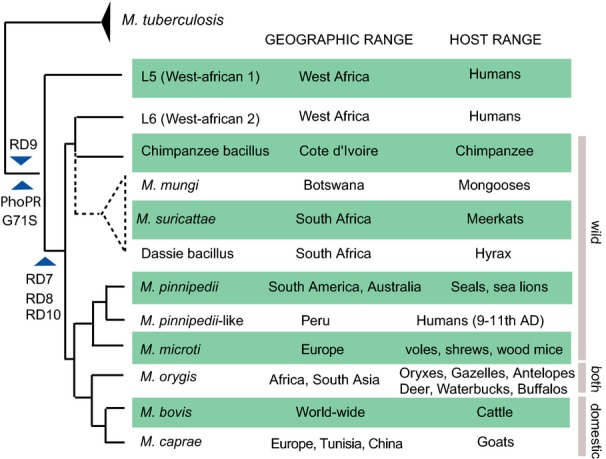
Simplified schematic illustrating the phylogenetic relationships of animal strains based on different phylogenies published previously ((31,40,49,51,52)). The length of the branches does not reflect real phylogenetic distances. Dashed branches represent putative relative relationships. The phylogenetic positions of genomic deletions discussed in the main text and of the mutations in the *phoPR* genes (54) are indicated. The geographical and host ranges of the animal lineages do not include reports from zoos, as those were considered incidental infections.

Some new insights into the genetic mechanisms that may explain why sustainable infections with animal-adapted strains only rarely occur in humans and the fact that *M. africanum* exhibits lower virulence than *M. tuberculosis sensu stricto* have recently been gained through some elegant work by Gonzalo-Asensio *et al*. (54). The authors showed that a mutation affecting the *phoPR* genes in the common ancestor of *M. africanum* Lineage 5 and Lineage 6 and of animal-adapted strains of MTBC, when transferred to a *M. tuberculosis sensu stricto* genetic background, lead to a decrease in virulence in both mice and human primary macrophages. PhoPR encode a two-component system regulating the synthesis and export of important *M. tuberculosis* virulence determinants such as LipF, ESAT-6, and lipids of the polyacyltrehalose and sulfolipid families (54,55). However, the data suggest that the secretion of ESAT-6 in *M. africanum* Lineage 6 and *M. bovis* is independent of PhoPR because of the loss of binding sites of the regulatory proteins EspR and MprAB that normally mediate ESAT-6 secretion in a PhoPR-dependent manner (54). This loss is caused by the deletion of RD8, suggesting that this deletion acted as a compensatory mechanism, restoring virulence in the common ancestor of *M. africanum* Lineage 6 and the animal-adapted lineages (54) (*Fig. *2). Strengthening this hypothesis, both *M. tuberculosis sensu stricto* and *M. canettii,* which represent a derived and the ancestor-like (see below) groups of the MTBC phylogeny, respectively, and which have no PhoPR mutations, regulate the secretion of ESAT-6 through PhoPR (54). Interestingly, the same PhoPR mutation is present in *M. africanum* Lineage 5, which has no deletion in RD8. Further work will tell whether an alternative compensatory change could have occurred in Lineage 5.

### Mycobacterium canettii

Within the genus *Mycobacterium*, the most likely bacteria to share the most recent common ancestor with MTBC are the so-called ‘smooth tubercle bacilli’ (STB), which include *M. canettii* (53,56,57). *Mycobacterium canettii* is formally part of MTBC, but unlike the other MTBC members described above, undergoes frequent horizontal gene transfer and inter-strain recombination (53,57). The genetic diversity between different strains of STB is comparable to that of other recombining bacteria, and much higher than the genetic diversity among the other MTBC lineages (53,57). Comparative genomics has revealed evidence of recombination between STB and other MTBC strains, although it remains to be demonstrated if these recombination events took place before or after the divergence of the different MTBC lineages (57–60). STB share an average nucleotide sequence similarity of over 95% and a high degree of synteny with the other MTBC members, justifying their inclusion in the complex (57). To date, only about 60 STB isolates have been reported, mainly from the Horn of Africa. STB seems to disproportionally affect expatriate individuals, children, and HIV co-infected TB patients (61). Furthermore, transmission of STB among humans has not been demonstrated; hence, these bacteria are probably acquired from the environment (62). However, no animal or environmental reservoir is currently known to harbor STB. Experiments in mice suggest both a lower virulence and shorter persistence during chronic infection with STB compared to H37Rv (57). Overall, the virulence phenotypes, the epidemiological characteristics, as well as certain genomic features such as the presence of different clustered regularly interspaced short palindromic repeats loci and associated *cas* genes (CRISPR-Cas), and the presence of CRISPR spacers matching prophages present in other mycobacteria (e.g. *M. marinum*) (61) are consistent with STB being environmental bacteria, only occasionally pathogenic in humans.

This finding is in sharp contrast to the other members of MTBC, which are obligate pathogens. The MTBC possibly evolved from a *M. canettii*-like ancestor, a strain with a loose ability to survive both in the environment and inside a host. The genetic changes associated with the transition to becoming a professional pathogen involved gene loses and gene acquisitions (57–59,63), and the diversification of gene families such as the *pe_pgrs* and *esx* gene families (64,65). Current examples of facultative intracellular bacteria with environmental reservoirs exist as the opportunistic pathogen *Listeria monocytogenes* illustrates (66). Between the *M. canettii*-like ancestor and the most recent common ancestor of all extant MTBC, the ability to replicate outside a host was lost and the ability to overcome the host defenses and to persist within host cells was acquired. This has come at the cost for the pathogen of harming the host (virulence), because at least some degree of pathology is essential for MTBC transmission to occur. This might have marked the beginning of the co-evolutionary association between *M. tuberculosis* and *Homo sapiens*. Notably, the trajectory toward host specialization has allowed this MTBC ancestor to spread all over the world and become one of the most successful human pathogens.

## The evolution of virulence in MTBC

In the process of becoming obligatory pathogens, the ancestors of MTBC must have evolved strategies to overcome the immune response of the host, to transmit between hosts, and to avoid becoming extinct in small human populations. The affected humans must have evolved counter strategies, because for transmission of MTBC to occur, human disease is necessary. Specifically, lung cavitation and increased sputum-smear positivity are associated with more efficient transmission of MTBC (67–71). Furthermore, crowding (i.e. increased host density) is known to enhance MTBC transmission (72). These characteristics suggest that MTBC virulence could have evolved under a trade-off: the pathogen has regulated its virulence to be able to persist and spread in human populations exhibiting increased densities and greater resistance or tolerance (5,73,74). There are two lines of evidence supporting this suggestion: one is the preferential association of particular MTBC lineages with their local human populations, which is highly suggestive of adaptation to their sympatric host populations, and the second is that different MTBC genotypes have evolved differences in virulence traits which might reflect the outcome of different strategies to persist in the specific human population they have evolved with. We now further discuss these points below.

### Local adaptation in human-adapted MTBC

If hosts are heterogeneous qualitatively (e.g. different genotypes) and quantitatively (e.g. abundance), temporal and spatial dynamics of host–pathogen associations are expected under a co-evolutionary process such that a snapshot in time or space will reveal that certain host genotypes are more susceptible to certain parasite genotypes (75,76). One example of such a dynamic is local adaptation, whereby a local pathogen population has a higher fitness in a local host (sympatric) than in a non-local host (allopatric) (75,76). Pathogens are expected to evolve quicker than their hosts due to their comparably reduced generation time, and thus tend to be ahead in the co-evolutionary process. However, the latter does not always hold true, depending on the specific characteristics of the local association (77). There is some evidence for local adaptation of human-adapted MTBC lineages to different host populations. Specifically, the geographic origin of a TB patient is in many cases a good predictor of the infecting MTBC lineage (78). This remains true in cosmopolitan areas such as San Francisco, London, and Montreal, where humans and bacteria intermingle (23,24,78,79). Furthermore, MTBC transmission was shown to be higher in sympatric host–pathogen associations than in allopatric combinations (24). Social factors such as preferential social mixing among ethnic groups are likely to account for some of these observations. However, there are indications that biological factors also play a role. For example, in a nation-wide molecular epidemiology study in Switzerland, it was found that recent transmission of sympatric MTBC strains was more likely than that of allopatric strains among European-born patients. Importantly, in contrast to HIV-negative TB patients, patients that were HIV-infected were more likely to be associated with an allopatric MTBC lineage (80). Moreover, this association became stronger with increased immune suppression as measured by reduced CD4^+^ T-cell counts (80).

Given that TB disease is necessary for MTBC transmission, what are the implications of local adaptation; are local MTBC strains more or less virulent in their local host population? One study in Texas has shown that different levels of pulmonary impairment could not be explained by differences in the causative MTBC lineages alone, but by the pathogen–host association; patients infected with sympatric MTBC lineages suffered less lung impairment than patients infected with allopatric lineages (81). Transmission was not measured in this study, so it is not possible to relate it to virulence. Nevertheless, it is likely that the different virulence characteristics of the different MTBC strains and lineages, such as their replication ability, the nature of immune responses elicited, and the duration of latency, are the result of fine-tuned adaptations to the particular local host population.

### Variation in virulence in human MTBC

The progression of TB in patients is largely determined by the response of the host immune system and environmental variables (17). In addition, mounting evidence points to an important effect of the MTBC genetic background (reviewed in (15,16)). The most abundant MTBC lineages in the world are representatives of the modern lineages, reflecting their evolutionary success. As already mentioned, these include the more transmissible and virulent strains of MTBC, some of which have been responsible for large outbreaks in different regions of the world, others associated with the emergence and spread of drug resistance. Representatives of Lineage 2 and Lineage 4 have generally a higher replication capacity in human macrophages and animal models of infection. There is also evidence that the modern Lineage 2, 3, and 4 elicit reduced and/or delayed pro-inflammatory immune responses (15,16). Lineages 2 and 4 have also been associated with higher transmission as measured by the number of secondary cases generated, reflecting a link between increased virulence in animal models and enhanced transmissibility in human populations (15,16). The high virulence of the modern lineages, in particular Lineages 2 and 4, has been hypothesized to have evolved as a response to the increasing size and density of human populations starting with the Neolithic Demographic Transition and the onset of agriculture (around 10 000 year ago), and later during the Industrial Revolution and the associated urban crowding (300–200 years ago) (26,29,32). In contrast, the much more geographically restricted *M. africanum* lineages show lower a prevalence compared to the modern lineages, even within their specific range of distribution in West African (30), with the possible exception of Guinea-Bissau (82). Moreover, there is indication that the prevalence of *M. africanum* lineages is decreasing in some parts of West Africa (82–84), possibly as a result of out-competition by *M. tuberculosis sensu stricto*. The *M. africanum* lineages are also known to be less virulent than modern strains in animal models (85), and *M. africanum*-infected patients have lower T-cell responses to ESAT-6, which is an important virulence factor in MTBC (86,87).

Latency is a hallmark of human TB (13,14). The majority of people infected with MTBC mount an effective adaptive immune response that controls the replication of the bacteria, leading to a persistent infection with no disease symptoms (88). Only a small fraction of latently infected individuals will re-activate to active disease, often several decades after initial exposure (14). By keeping MTBC replication in check, the host avoids MTBC virulence. However, latency could also have evolved as a strategy for the pathogen to ensure transmission and avoid extinction in small host populations (89). Supporting this view, MTBC evolved several mechanisms to survive during latency (90,91). Mathematical models suggest that optimal levels of persistence of MTBC in human populations would be achieved with a latency period and the ones currently observed in clinical settings, suggesting that the human immune system keeps the duration of latency and activation rates under their evolutionary optimum (92). Latency seems thus neither uniquely a property of the host or the pathogen, and it has most likely been shaped by the interactions between the two. This is supported by the observation that in the Gambia, patients infected with *M. africanum* Lineage 6 were less likely to progress to active TB than those infected with other MTBC lineages (93). Indeed, the rate of progression to active disease was 5 times higher for Lineage 2 than for *M. africanum* Lineage 6 (93).

## Host–pathogen molecular interactions

It is well established that human genetic factors influence clinical TB phenotypes (94,95). Family aggregation and twin studies have revealed a clear genetic basis for susceptibility to TB infection and disease (reviewed in (95)). Furthermore, susceptibility to infection correlates with human ancestry (95–97). However, identifying the human genetic variants underlying the various clinical manifestations of TB has had limited success (reviewed in (94,95)). For example, polymorphisms in the gene *NRAMP1/SLC11A1* have been associated with pulmonary TB (98). Still, despite a strong support for the role of this gene in TB, its effect is highly heterogeneous across different human populations (98–100).

Much of the difficulties in uncovering the human genetic variants underlying responses to MTBC are due to the inability in unambiguously defining and quantifying relevant phenotypes (11,13,14). In addition, age, nutritional status, and co-infections can modulate the clinical presentation of TB (101). Finally, co-evolution may also account for some of those difficulties (4,7). As introduced before, in a co-evolutionary host–pathogen association, the outcome of the interaction between the partners depends on their respective genotypes (5–8). In TB, there is now increasing evidence that the interaction between bacterial and human genetic loci influence clinical phenotypes. For example, one study found that in a Vietnamese population, carrying the T597C allele of the *Toll-like receptor 2* gene (*TLR2*) was associated with infection by Lineage 2 (102). Another study from Ghana found that the variant G57E of the *Mannose-binding Lectin (Protein C) 2* gene (*MBL2*) was associated with TB caused by *M. africanum* as opposed to *M. tuberculosis sensu stricto* (103). Also in Ghana, the variant 261TT of the *Immunity-related GTPase M* gene (IRGM) was protective against TB caused by Lineage 4, but not against disease caused by other MTBC lineages (104). A recent study reported on the association between different HLA class I types and disease caused by different MTBC strain families in a South African colored population (105). These results suggest that at least to some extent, TB clinical phenotypes can be explained by the interaction between human and MTBC genetic variation. Thus, new insights will be gained when MTBC strain information is accounted for in human genetic studies of TB. Indeed, a recent study in HIV has used a ‘genome-to-genome’ approach to successfully identify interacting molecular partners in the virus and the human host (106). Co-evolution is thus likely to have important implications for our understanding of the association between humans and MTBC and, more than just stimulating interesting academic discussions, can guide our research toward better tools to control the disease. One particular discussion among academic parties concerns the age of the association between humans and MTBC, which we address in the following section.

## The age of the association between MTBC and humans

Particular questions related to the age of this association include how and when the different MTBC lineages emerged and diversified. Moreover, did enough time elapse for differences in virulence phenotypes to evolve, and does the age of MTBC explain its low genetic diversity compared to other bacteria? Many studies have speculated on the age of the most recent common ancestor (MRCA) of MTBC, but only recently has the subject been addressed in a quantitative way. Four studies have used DNA diversity to infer the age of MTBC (32,40,29,107) (*Table *1), while two other studies have inferred the age of particular MTBC variants (23,108). However, all of these estimates remain controversial. We review here some of these estimates and contrast them with the available archeological evidence. An important point to keep in mind is that using DNA sequences for these types of inferences can only tell us about the MRCA of the DNA sequences used in the analysis. As pointed out by Smith *et al*. (22), if for example the Beijing family had taken over all the remaining lineages in the world, the MRCA of all contemporary MTBC would be a Beijing strain.

### What do DNA sequences tell us about the age of MTBC?

Dating the age of bacteria relies on a molecular clock (*Table *1), i.e. a way of estimating how much time has elapsed since the corresponding DNA sequences diverged. Dating the MTBC phylogeny has several complications, which the studies we review here tried to circumvent in different ways. It is now widely accepted that there is no universally applicable molecular clock in bacteria, and that the molecular clock of different bacterial species and lineages can differ by orders of magnitude (109,110). The first study attempting to determine a molecular clock for MTBC was by Wirth *et al*. (32), using microsatellite-like loci with variable numbers of tandem repeats. The common ancestor of MTBC was dated at around 40 000 years ago, and the data supported the divergence of two main lineages around 20 000–30 000 years ago, which co-migrated with modern humans and spread through Africa and Eurasia. The limitation of this study lies on the genetic markers used. Microsatellites are excellent genotyping makers for epidemiological purposes because of their fast rate of evolution, but their molecular evolution patterns cannot be easily extrapolated to the genome as a whole. Therefore, they are of limited use for phylogenetic inferences (111).

**Figure 3:**
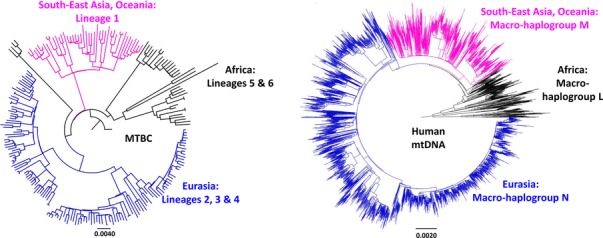
Comparison of the MTBC phylogeny based on 220 genomes (left) and a phylogeny derived from 4955 mitochondrial genomes representative of the main human mitochondrial haplogroups (right). The color coding highlights the similarities in tree topology, and geographic distribution between MTBC strains and the main human mitochondrial macrohaplogroups (black, African clades: MTBC Lineages 5 and 6, human mitochondrial macrohaplogroups L0–L3; pink, Southeast Asian and Oceania clades: MTBC Lineage 1, human mitochondrial macrohaplogroup M; blue, Eurasian clades: MTBC Lineages 2, 3, and 4, human mitochondrial macrohaplogroup N). MTBC Lineage 7 has only been found in Ethiopia, and its correlation with any of the three main human haplogroups remains unclear. Figure from Comas *et al*. (29).

**Figure 4:**
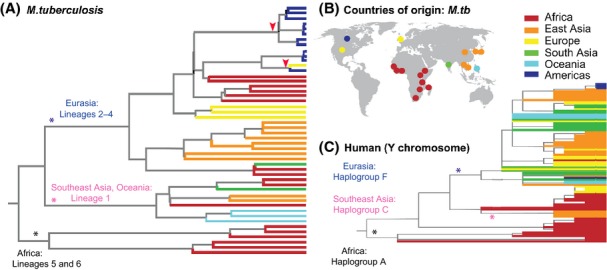
Geographic and genetic structure of a global sample of MTBC genomes. Adapted from Pepperell *et al*. (107). (A) Maximum clade credibility phylogeny inferred from genome-wide MTBC SNP data. Tips are colored by the geographic origin of the MTBC isolate (see key). Modifications: the lineage nomenclature (indicated with an asterisk) and color codes used by Comas *et al*. (29) was added to the original tree; the red arrows indicate the calibration points used for dating the most recent common ancestor of MTBC. (B) Countries of origin for MTBC isolates used in this study are shown as colored dots on the global map. One dot is shown per country but some countries were represented by >1 MTBC isolate. Colors correspond to global regions (see key). (C) Phylogeny of global human populations based on Y chromosome data (120). Tips are colored according to the same scheme as the MTBC phylogeny (A). Modifications: the names of the relevant human Y haplogroups were added (indicated with an asterisk) and color coded as in Comas *et al*. (29).

More reliable phylogenetic calibration points can be obtained by dating nodes or tips of a phylogenetic tree using ancient bacterial DNA (aDNA), but this was until very recently (see below) not possible for MTBC, because only small fragments of aDNA with little or no phylogenetic information were available. In the absence of calibration points, another possibility is to estimate mutation rates experimentally (112) or from epidemiological outbreaks (113). Importantly, the relationship between time and substitution rate is poorly understood in MTBC. In other words, the way mutations accumulate during short periods of time, say years to a few decades, is not a good approximation for how mutations accumulate over longer time periods relevant for evolutionary processes (109). Thus, extrapolating from short-term mutation rates to long-term substitution rates is questionable. Moreover, the short-term mutation rates themselves are debatable because of the uncertainty of the *in vivo* generation time and the strong genetic drift associated with population bottlenecks (further discussed below). One alternative way to calibrate phylogenetic splitting events, for which bacterial divergence is strictly a consequence of co-divergence with its host, is to use the host fossil record to date these events. This approach has been particularly powerful in the case of vertical transmitted endosymbiotic bacteria and their insect hosts (114). A similar approach has been used successfully to estimate the age of the association between *Helicobacter pylori* and humans. Even though *H. pylori* is horizontally transmitted, transmission occurs often between close relatives, i.e. from a mother to her newborn child, hence approaching ‘vertical’ transmission (115). Recently, Comas *et al*. (29) used such an approach to infer the age of the MCRA of MTBC. The starting observation was the striking similarity between the MTBC phylogeny based on 220 full genome sequences representing the global diversity of human-adapted MTBC, and a human phylogeny based on 4955 mitochondrial genomes representing the main human mitochondrial haplogroups (*Fig. *3). The authors observed that important branching events coincided in both phylogenies. Specifically, the earliest branching clades had an African origin in both cases, and the branching of Lineage 1 and the Eurasian Lineages 2 to 4 mirrored the main human mitochondrial haplogroups M and N associated with the earliest Out-of-Africa migration of modern humans (*Fig. *3). In addition, there were strong quantitative associations between the most frequent MTBC lineages and the most common mitochondrial haplogroups from the same countries. The authors interpreted these observations as evidence for co-divergence, and thus used different events in human evolution to date the MTBC phylogeny (29). Specifically, the coalescent time (*Table *1) for the basal MTBC Lineages 5 and 6 was calibrated using three alternative key points in human evolution; the emergence of anatomically modern humans (approximately 185 000 years ago), the emergence with the human mitochondrial DNA haplogroup L3 (approximately 70 000 years ago), and the emergence associated with the beginning of the Neolithic Demographic Transition (approximately 10 000 years ago). In this way, several dates were obtained for the different branching points in the MTBC phylogeny separating the seven known MTBC lineages. Among these three possible scenarios, the one yielding a coalescent time for Lineages 5 and 6 of 70 000 years was considered the most likely, as the MTBC lineage splits could be related to known human migratory events and human archeological evidence. For instance, the first split leading to Lineage 1 was estimated at around 67 000 years ago, coinciding with the first wave of human migrations Out-of-Africa (29). The split leading to Lineages 2 and 4 was estimated at 30 000–46 000 years ago and at 32 000–42 000 years ago, respectively, which correlates well with the first archeological evidence for the presence of modern humans in Europe and East Asia, respectively (116,117). The age of the MRCA of all members of the complex was thus estimated at 73 000 years (range 50 000–96 000 years ago). Although only based on correlations, the scenario proposed by this study offers plausible explanations for the phylogeographic structure observed in MTBC (*Fig. *1). It has one important limitation, which is the *a priori* assumption of co-divergence between the MTBC and humans. Yet, mirrored phylogenies between hosts and their pathogens are not necessarily the result of co-divergence, as switching between closely related hosts and extinctions of certain pathogen lineages can lead to similar phenomena (118,119). As outlined above, extant human-adapted MTBC are obligate human pathogens not known to sustainably infect any other host species and with no known environmental reservoir, but whether that has always been the case throughout the evolution of MTBC is unknown. Thus, for horizontally transmitted pathogens such as MTBC, ideally, the coinciding splitting events of both the host and the pathogen lineages should be demonstrated independently. This has been tested in the work by Pepperell *et al*. (107), in which a phylogeny of global human populations based on the Y chromosome (120) and a phylogeny of 48 global MTBC genome sequences were compared. Unlike Comas *et al*. (29), these authors did not find evidence for congruence between the human and the MTBC tree topologies (*Fig. *4). It is known that human phylogenies based on Y chromosomes and mitochondrial DNAs can provide evidence for different human evolutionary histories even in the same geographic regions, but broad features of human evolution are consistent between the two markers (120). Accordingly, it appears that in general, the similarity between the topologies of the human phylogeny based on Y chromosomes and MTBC is not as negligible as originally suggested by Pepperell *et al*. (107) (*Fig. *4*A and C*). In *Fig. *4*A*, we added to the internal nodes of the MTBC phylogeny by Pepperell *et al*. (107), the origin of the most recent common ancestor of each main lineage as determined by Comas *et al*. (29) (*Fig. *3). This is justified, as patterns consistent with co-divergence are more likely to be found when considering major splits that happened in a more distant evolutionary past rather than considering more recent splitting events such as the ones reflected on the tips of the phylogeny. For example, Lineage 2 strains can be found in South Africa; however, because these strains were most likely introduced by recent (in evolutionary time) human migrations from Asia (121), it is unlikely that a signature of co-divergence will be observed between South African Lineage 2 strains and South African human populations. In addition, based on the original publication (120), we indicated in the human phylogenetic tree the Y haplogroups relevant for this discussion (*Fig. *4*C*). We highlight here what we consider to be congruent among *Figs *3 and 4: (i) a strong concordance in the split of the early branches that are restricted to Africa (Lineages 5 and 6 versus Y chromosome haplogroup A and mitochondrial macrohaplogroup L); (ii) concordance in the split that originated MTBC Lineage 1 and the Y chromosome haplogroup C and the mitochondrial macrohaplogroup M that are found in East Asia, South Asia, and Oceania (120); and (iii) the split of both the Y chromosome haplogroup F and the mitochondrial macrohaplogroup N is in concordance with the split of the modern Eurasian MTBC Lineages 2, 3, and 4.

Important differences between the results of Comas *et al*. (29) and those of Pepperell *et al*. (107) reside in the approach taken to infer the age of the most recent common ancestor of MTBC. In Pepperell *et al*. (107), the rate of evolution of MTBC was determined using two calibration points which are marked in *Fig. *4. The first calibration point was applied to eight MTBC strains isolated from Western Canadian Aboriginal TB patients, strains which according to historical records have been introduced from Europe between 1710 and 1870 C.E. through the fur trade (122). The second calibration point was applied to the divergence point between the laboratory strain H37Rv and a closely related Canadian *M. tuberculosis* clinical isolate. The date for the divergence between these two isolates was set at 1905, the date of the original isolation of H37Rv from a patient in New York (123). Using this approach, the authors estimated a substitution rate of 1.3 × 10^−7^ substitutions/site/year, which is much higher than the one estimated by Comas *et al*. (2.58 × 10^−9^ substitutions/site/year), and close to the mutation rate (0.2–0.5 SNPs per year) inferred from molecular epidemiology studies (113,124,125). This higher substitution rate translates necessarily in a much more recent common origin for all MTBC lineages (*Table *2). To test more formally for co-divergence, Pepperell *et al*. (107) correlated divergence time among different MTBC estimates with human estimates of genetic distance between continental population and concluded that this correlation was too low (*r*^2^ = 0.46) to support co-divergence. We agree this is an important analysis, but it should be repeated using more isolates spanning more of the global diversity of MTBC.

**Table 2:** Comparison of different dating scenarios for the evolution of MTBC

Coalescent times	Comas *et al*. (29)	Pepperell *et al*. (107)	Bos *et al*. (40)
MRCA of MTBC	73 000 (50 000–96 000)	n.r.	(2962–5339)
Lineage 5 and 6	70 000 (48 000–88 000)	2190 (1331–3142)	(2792–5025)
Lineage 1	67 000 (46 000–88 000)	2190 (1331–3142)	(2765–5105)
Lineages 2–4	46 000 (31 000–61 000)	1347 (830–1907)	(1779–3422)

Coalescent times (*Table *1) are given in thousands of years. The median and/or the 95% HPD (highest probability density) intervals are indicated; n.r., non-reported.

In another approach, Bos *et al*. (40) used the *M. pinnipedii*-related aDNA samples from Peru mentioned above (radiocarbon dated to between AD 1028 and AD 1280) to calibrate a global MTBC phylogeny. The substitution rate obtained was 4.6 × 10^−8^ substitutions/site/year, again much higher than the estimate by Comas *et al*. (29) and closer to the one by Pepperell *et al*. (107) and the various epidemiological studies (113,124,125). The MRCA of MTBC was inferred at 4449 years before present, similar to the estimate by Pepperell *et al*. (107) (*Table *2). The same work (40) corroborated these recent time estimates using an independent calibration based on a 18th century Hungarian mummy from which MTBC DNA was isolated and genome sequenced (126). The year of the supposed death of the individual carrying the bacillus from which the DNA was isolated (1797 AD) was used (126). The substitution rate was only slightly higher (7.07 × 10^−8^ substitutions/site/year) than the one obtained with the Peruvian aDNA (40).

The estimates by Pepperell *et al*. (107) and the ones obtained with the Hungarian mummy DNA (40) have some technical limitations. Substitution rates (*Table *1) are known to be time-dependent (127,128). Estimates based on transient mutations (i.e. polymorphisms) such as the ones obtained in epidemiological contexts or by dating the tips of phylogenetic trees built with extant or very recent strains in evolutionary terms, are necessarily higher than estimates based on fixed mutation (i.e. substitutions) because a high proportion of polymorphisms is eliminated by natural selection and genetic drift over time (26,107). The relative contribution to the evolution of MTBC of natural selection and genetic drift is discussed further below. The important point in the context of dating MTBC phylogenies is that in MTBC, we do not know how far back in time polymorphisms can persist. Using the approximately 1000-year-old DNA to calibrate the MTBC phylogeny should lessen this problem (40). However, the similarity of the substitution rate estimates obtained using this approach and the ones obtained through epidemiological studies is not very reassuring. An additional problem is that the different MTBC lineages most likely evolve at different rates (40), meaning that the molecular clock inferred with the Peruvian aDNA does not necessarily reflect the molecular clock of the different MTBC lineages. To resolve more confidently the age of the most recent common ancestor of MTBC and of its main lineages, other calibration points, preferably based on aDNA from older MTBC strains will be necessary.

**Figure 5:**
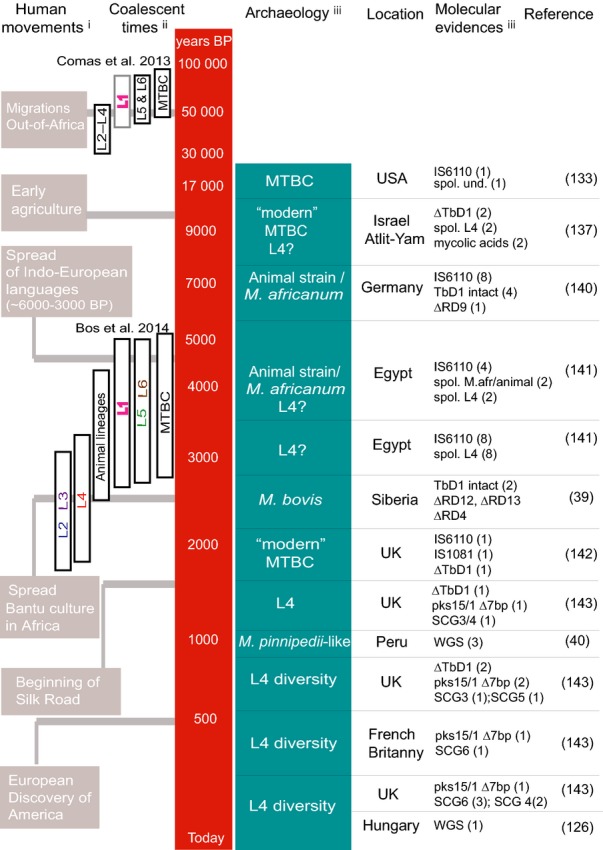
Summary of the most important archeological findings and molecular evidence indicating the presence of MTBC in human remains. All studies reported MTBC in human skeletons or mummies except in Rothschild *et al*. (133). (i) Human movements possibly involved in the expansion of MTBC; (ii) Intervals for coalescent times (95% HPD) concerning the MRCA of different MTBC lineages obtained from the two most discrepant inferences (40,29); (iii) Interpretation of both archeological and biomolecular findings. For a better understanding of the molecular typing techniques see Coscolla and Gagneux (15). For each molecular marker, the number of archeological specimens containing DNA with that specific marker is indicated within parentheses. BP, before present; spol., spoligotyping pattern; und., undefined; WGS, whole-genome sequencing.

The estimates by Bos *et al*. (40) suggest that the ancestors of all current MTBC lineages could have originated as recently as 3000 BCE to 2000 BCE and that the common ancestor of all modern lineages appeared only between 1500 BCE to 200 CE (*Table *2, *Fig. *5). How then to explain the similarity between the human and the MTBC phylogenies as described in Comas *et al*. (29)? Moreover, how did the modern lineages spread Out-of-Africa to the rest of the world? Did they emerge as one Out-of-Africa lineage, spreading and diversifying through the Indo-European migrations that between 4000 and 1000 BCE connected Asia Minor, Europe, Central Asia, and India (129)? Could the human movements associated with the Silk Road linking different parts of Asia, India, and the Mediterranean region have spread Lineage 2 and Lineage 3 strains (130) (*Fig. *5)? These more recent estimates also imply that MTBC has diversified and adapted to a whole range of distinct mammalian species (*Fig. *2) during a period of only about 4000 years. Archeology from human remains coupled with molecular biological analyses of mycobacterial aDNA has contributed significantly to our understanding of the association of MTBC and ancient human populations (131,132). We briefly summarize some of those findings next.

### What does the archeological evidence tell us?

In *Fig. *5, we summarize the most significant archeological contributions based on bone lesions consistent with TB and molecular evidence for the presence of MTBC in archeological specimens. We briefly discuss here the inconsistencies between the archeological findings and the age estimates based on DNA sequence divergence discussed above. The estimates by Comas *et al*. (29) predate all archeological evidence known to date and cannot thus be refuted. In contrast, there are inconsistencies with the dating estimates obtained by Bos *et al*. (40) and Pepperell *et al*. (107). The oldest known biomolecular evidence for MTBC dates to about 17 500 years before present and was found in a bison bone discovered in Wyoming, USA (133) (*Fig. *5). This finding predates the earliest estimates for the age of the MRCA of MTBC by Bos *et al*. (40) (*Fig. *5). The molecular evidence indicative of MTBC aDNA was the amplification of IS*6110* and presence of spoligotyping patterns, both of which are specific to MTBC today (134). However, the insertion element IS*6110* is a mobile genetic element, we can therefore not be sure of the specificity of this element in the past (135). Similarly, the genomic region used for spoligotyping is known as the CRISPR-Cas region which is prone to horizontal gene transfer in other bacteria (136).

Another archeological finding inconsistent with the age of the MRCA of all modern lineages inferred by Bos *et al*. (40) comes from one report on TB caused by modern strains of MTBC in an approximately 9000 years old human skeletons from Israel (137) (*Fig. *5). The strain in question harbored the TbD1 deletion, spoligotyping patterns could be obtained, and the presence of mycolic acids was also detected. However, the authenticity of some of this material has been the subject of dispute (138,139). Generally, studies using aDNA are prone to contamination, and many precautions need to be taken for generating convincing evidence (131).

The earliest estimate for the age of the MRCA of all animal-adapted lineages of MTBC was set at around 4000 years by Bos *et al*. (40) (*Fig. *5). The oldest archeological evidence for an animal MTBC strain has been reported from a human skeleton from Germany, which presented evidence of TB caused by a strain with an RD9 deletion dated to around 7000 years ago (140) (*Fig. *5). Data from other human skeletons from the same site also suggested that as early as 5000 BCE, MTBC was already infecting humans in Central Europe (140) (*Fig. *5), an observation that does not fit with the age of modern MTBC lineages proposed by Bos *et al*. (40). Alternatively, other MTBC strains might have existed in Europe and been later displaced later by modern strains. Modern strains might have been already present 4000 years ago in Egyptian mummies (141), and these strains were also detected as causing TB in the United Kingdom during the Iron Age 2200 years ago (142) (*Fig. *5). Another study suggests that *M. bovis* had reached Siberia by 2000 years ago (39). Interestingly, more recent studies have achieved more precise classification of ancient MTBC strains (40,126,143), which indicates that, as mentioned before, strains similar to extant *M. pinnipeddi* infected humans in Peru around 1000 years ago (40), and around the same time, different genotypes of Lineage 4 were present in the United Kingdom (143) (*Fig. *5). The relative abundance of MTBC DNA in archeological human remains, together with our increased ability to sequence reliably aDNA, will certainly shed further light on the co-evolutionary history of MTBC and humans in the near future. A complementary approach to study the co-evolutionary history of humans and their pathogens is to determine how the genetic variation that characterizes the extant populations of both has been shaped over time (4,7).

## Co-evolutionary genetics: the MTBC perspective

In a co-evolutionary association, molecular interactions between the host and the pathogen are expected (7), such as the ones involving host immune receptors and pathogen surface proteins. For example, if a mutation encodes a certain molecular variant that allows a pathogen to avoid host recognition, the frequency of that mutation will increase. Likewise, mutations in a host leading to better recognition of that pathogen variant will be selected. Over evolutionary time, such dynamics lead to distinctive signatures of selection in the genetic diversity of the affected loci of both the host and pathogen populations (4,7). The interpretation of these signatures can give us insights into which loci are affected and how they evolve. However, natural host–pathogen associations show us that co-evolving hosts and pathogens have many additional characteristics which complicate our assessment of the relevant loci involved in these interactions, and the relevant time and geographic scales to be considered (3,7). For example, humans have long generation times compared to their pathogens and the molecular variation relevant for the recognition of the pathogen may be somatically generated *de novo* (adaptive immune system) and will thus not be encoded in their DNA. Alternatively, pathogens may evolve strategies other than avoiding being recognized by the host. In MTBC, this seems to be the case as suggested by one study which studied variation in 491 experimentally confirmed T-cell epitopes in representatives of several lineages of the complex (27). Surprisingly, that study found that the large majority of these T-cell epitopes had high sequence conservation, indicating that there is a strong selection pressure to keep these T-cell epitopes unchanged (27,107). One possible reason for this high conservation of T-cell epitopes is that MTBC might benefit from being recognized by T cells because, as referred above, the microbe depends on an efficient host immune response leading to increased lung pathology to transmit to new hosts (27). Support for this view comes from the observation that HIV co-infected TB patients tend to generate fewer secondary cases compared to HIV uninfected patients (144); this is particularly true for HIV/TB patients with low CD4^+^ T cells (145). Interestingly, sequence variation in T-cell epitopes is also low in *M. canettii* (57) and in BCG strains, which evolved during *in vitro* passage (10), suggesting that recognition by T cells is probably not the only reason underlying epitope conservation in MTBC.

In addition to mutations causing polymorphisms in populations, duplication, and expansion of gene families has evolved in almost all organisms as a way to generate genetic diversity (146). In particular, multi-copy gene families have evolved as a strategy to generate antigenic variation in different pathogens; classical examples include the *var* genes of *Plasmodium falciparum* and *pil* genes of *Neisseria meningitis* (147). In MTBC and other virulent mycobacteria, the *pe_pgrs* genes underwent extensive duplication (148). Moreover, these genes harbor higher diversity than other parts of the MTBC genome (149,150). However, similar to the hyperconservation of T-cell epitopes encoded by other parts of the MTBC genome (27), the regions of *pe_pgrs* genes that encode T-cell epitopes are mostly confined to the PE domains, which exhibit high sequence conservation when comparing different clinical strains of MTBC (10). Thus, the diversity of *pe_pgrs* genes cannot be explained by evasion of T-cell recognition. Perhaps, the importance of the genetic diversity of *pe_pgrs* genes lies not so much on the diversity generated at the population level but rather on the diversity generated within each bacterial cell, for instance by differential expression of these genes under different conditions. Another possibility is that the diversity of *pe_pgrs* genes might be driven by host antibody recognition (10). In summary, there is currently no evidence for immune evasion through antigenic variation in the genomes of MTBC.

Is there any other evidence of adaptation in MTBC genomes? The emergence of drug resistance clearly illustrates that MTBC has the potential to adapt. Yet, except in the context of drug resistance, the current evidence of ongoing adaptive changes in MTBC is scarce. One important limitation to adaptive evolution in MTBC might be the lack of recombination and horizontal gene transfer (151). Recombination reduces the extent to which the effect of natural selection on new mutations is reduced by the effect of selection imposed by the genetic background (background selection, *Table *1), i.e. recombination can separate advantageous and neutral mutations from harmful mutations (152). In non-recombining chromosomes, advantageous mutations might get ‘trapped’ with mutations that are slightly deleterious, in which case, selection on the advantageous mutation has to be strong for it to be selected (152).

Nevertheless, evidence for adaptation is emerging as more full genomes of MTBC clinical strains become available for comparative analyses. A recent analysis by Osorio *et al*. (153), reported two genes unrelated to drug resistance showing evidence of positive selection in MTBC. The study by Gonzalo-Asensio *et al*. (54) described above on the molecular basis of virulence in Lineage 6 and animal-adapted lineages presents compelling molecular evidence for adaptation. From a more speculative point of view, it is also plausible that MTBC and humans might be close to an evolutionary ‘optimum’. This would imply that the human-adapted MTBC lineages are generally well adapted to their host and *vice versa*. If so, most mutations responsible for host adaptation would already have reached fixation. New mutations will mostly be neutral or slightly deleterious and hence assume the form of transient polymorphisms, which end up disappearing from the population over time. Such a scenario has been suggested for other genetically monomorphic bacterial pathogens (154). Thus, most of the main reciprocal adaptations between MTBC and humans would have happened in the distant evolutionary past. This does not mean that MTBC and humans do not co-evolve anymore, but adaptation now might involve much smaller effects. Some indirect support for this idea comes from two studies of ancient and historical patterns of alleles conferring human resistance to TB. One concluded that the dramatic decline of TB in Europe starting in the mid-nineteenth century, long before modern interventions such as BCG vaccination and antibiotic treatment were introduced, cannot be the result of selection for higher genetic resistance to TB (155). Contrastingly, and supporting the view that the bulk of co-evolution occurred in the evolutionary past, a study by Barnes *et al*. (156), suggested that the time since ancient urbanization in the Old World (the earliest settlement considered was 6000 BCE) can explain TB genetic resistance in today's urban human populations.

Alternatively, it has been hypothesized that infection by MTBC might have been beneficial to early human populations during times of nutrient shortage (157) and/or by providing some level of immunological protection against other pathogens (29). It is provocative to propose that a pathogen killing 1.3 million people every year might have reached some sort of evolutionary optimum or might even offer a net benefit to infected humans. At the same time, it is clear that only a small proportion of the more than 2 billion individuals estimated latently infected individuals whose immune system is able to control the infection will never die of or exhibit any symptoms of TB.

### Genetic drift versus adaptation

It has been proposed that the evolutionary trajectory of new mutations in MTBC might be mostly governed by genetic drift, negative selection, and background selection (26,60,107) (*Table *1), except in context of drug treatment or HIV co-infection (158). Generally, due to their (on average) negative functional effects, non-synonymous single nucleotide mutations are removed more efficiently by natural selection than synonymous mutations (generally considered to be neutral). In MTBC, a high proportion of non-synonymous mutations are maintained across almost all genes categories relative to the corresponding synonymous mutations (26,107). Moreover, close to half of these non-synonymous mutations have been predicted to impact protein function, presumably mainly in a negative way (26,159). These phenomena are consistent with an important role for genetic drift (i.e. ‘chance’, *Table *1) in the evolution of MTBC. This is likely, given the strong sequential population bottlenecks (*Table *1) during patient-to-patient transmission, where new infections are initiated by only a single or a few bacterial cells (160). The effects of genetic drift on the evolutionary trajectory of mutations can be very strong, because drift reduces the effective population size of a population (the number of individuals that contribute to the progeny of the following generation), decreasing the efficiency of natural selection in removing deleterious mutations. Moreover, mutations that are neutral or have only a small advantageous effect on fitness can be lost by chance. The notion that genetic drift plays an important role in MTBC is not to say that selection is completely absent. Indeed, as discussed above, some signatures of positive selection (i.e. adaptation) have been observed.

## Concluding remarks and outlook

The observations we reviewed here favor the hypothesis of co-evolution between humans and certain MTBC lineages. Humans differ in their genetic susceptibility to TB, and the genetic diversity in MTBC is also increasingly recognized as a factor influencing the outcome of TB infection and disease. Moreover, recent studies have reported interactions between human genetic diversity and bacterial diversity, reinforcing the view that in addition to environmental variables, TB susceptibility varies as a function of both the human and MTBC genotype. The age of the association between humans and MTBC is subject to intense debate, but irrespective of the exact duration of this association, different phylogenetic lineages of MTBC are associated with different geographic regions, and possibly adapted to specific human populations. In addition, some lineages seem to behave in a generalist way, while others appear to be specialists restricted to a smaller group of human host populations.

Co-evolution is defined as reciprocal adaptive changes in interacting host and pathogen genetic loci. Conceptually, these interacting loci can be seen as in ‘conflict’ with each other (161). Identifying these conflicting host and pathogen loci could shed new light onto the host–pathogen interaction by identifying the actual molecular determinants involved in this interaction. Moreover, from a vaccinology point of view, these conflicting loci might be attractive new vaccine targets, because the host immune responses they elicit will most likely be detrimental to MTBC. This is in contrast to the highly conserved T-cell epitopes that elicit immune responses that might offer a net benefit to the bacteria (27). Hence, in addition to being an interesting academic subject of discussion, a better understanding of the co-evolutionary history of MTBC and *H. sapiens* could be exploited in vaccine development. For example, as illustrated in the recent genome-to-genome study of HIV mentioned above (106), a similar approach could be used to identify new protective antigens for TB vaccines.
